# Short-Term Effects of Capacitive and Resistive Electric Transfer Therapy and Static Splinting in the Management of Trigger Finger: A Non-Randomized Clinical Study

**DOI:** 10.3390/life16010030

**Published:** 2025-12-25

**Authors:** Larisa Ryskalin, Federica Fulceri, Paola Soldani, Andrea Poggetti, Paolo Annoscia, Chiara Marinelli, Giulia Ghelarducci, Marco Gesi

**Affiliations:** 1 Department of Translational Research and New Technologies in Medicine and Surgery, University of Pisa, Via Roma 55, 56126 Pisa, Italy; larisa.ryskalin@unipi.it (L.R.); federica.fulceri@unipi.it (F.F.); paola.soldani@unipi.it (P.S.); 2 Hand and Reconstructive Microsurgery Unit, Azienda Ospedaliero Universitaria Careggi, 50134 Florence, Italy; poggetti.andrea@gmail.com; 3 Hand and Reconstructive Microsurgery Unit, Azienda Ospedaliero Universitaria-University of Pisa, Via Roma 67, 56100 Pisa, Italy; p.annoscia@gmail.com; 4 Hand Unit, San Rossore Clinical Center, 56122 Pisa, Italy; c.marinelli1994@gmail.com (C.M.); ghelarduccigiulia1@gmail.com (G.G.)

**Keywords:** stenosing tenosynovitis, adult trigger finger, triggering, orthosis, diathermy, TECAR therapy, pain management, clinical effectiveness

## Abstract

Trigger finger (TF) is a common debilitating hand disorder that often results in pain and functional limitations. Currently accepted conservative treatments include splinting, nonsteroidal anti-inflammatory drugs, and corticosteroid injections. Recently, transfer energy capacitive and resistive (TECAR) therapy is emerging as a promising intervention for its potential to enhance microcirculation, reduce pain and inflammation, and stimulate tissue regeneration in various musculoskeletal conditions. However, its effectiveness for TF remains unclear. This study aims to compare the outcomes between TF conservative management with splinting alone, TECAR therapy alone, and the combined approach. Twenty-one fingers from 16 patients were included, with outcomes measured in terms of pain intensity, Green’s TF classification, frequency, severity and functional impact of triggering, and Quick-Disabilities of the Arm, Shoulder, and Hand. Assessments were conducted at baseline, at the fourth and eighth weeks after the initial treatment, and at one-month follow-up. Although all groups showed beneficial effects in all outcomes from baseline to the follow-up, the combined therapy group demonstrated statistically significant, quicker, and higher magnitude improvements in all clinical parameters compared to the single-treatment groups. By combining TECAR therapy with conventional splinting, TF patients may experience faster pain relief and functional recovery. Thus, integrating TECAR therapy into rehabilitation programs may represent a valuable opportunity for enhancing pain management and recovery in TF patients.

## 1. Introduction

Trigger finger (TF), also known as stenosing tendovaginitis or stenosing flexor tenosynovitis, is a common atraumatic and debilitating hand condition characterized by pathological thickening of the flexor tendon or its sheath, leading to nodule formation and impaired tendon gliding [1,2,3,4,5]. Clinically, patients typically experience a clicking sensation during active motion of the digit, accompanied by sharp pain, which may progress to painful locking of the finger in a flexed position, with inability to extend it except with the assistance of the contralateral hand, as occurring in more advanced stages [6,7]. The first annular pulley (A1), located proximal to the first metacarpo–phalangeal (MCP) joint, is by far the most affected pulley in this condition [8,9], with the ring finger and the thumb being reported as the most commonly involved digit [5,10,11]. Furthermore, multiple-digit involvement is not unusual [1]. TF shows a bimodal incidence pattern, with a first peak occurring in children under 8 years of age (pediatric TF, also known as Notta’s disease), and a second, more common peak in adults, between the fifth and sixth decades [1]. While in the pediatric population, the TF demonstrated an equal distribution in both genders, in adults, women are reported to have a sixfold increased risk compared to men [5,9,12].

Although its exact etiology remains unknown, TF is generally considered a degenerative condition associated with repetitive finger movements, local occupational microtrauma, and increased compressive forces in the A1 pulley [5]. These factors may explain the higher incidence in the dominant hand, although evidence regarding the role of occupational exposure remains inconclusive [2,5,13,14,15,16].

Treatment for TF can be either conservative or surgical (i.e., open, percutaneous, and endoscopic releases) [17,18,19]. Nonsurgical interventions are usually recommended as the first-line care, and include custom-made orthotics, corticosteroid injections, nonsteroidal anti-inflammatory drugs (NSAIDs), and extracorporeal shockwave therapy [18,20,21,22]. Although several conservative treatments for TF have been described in the literature, the most effective is still debated.

Current scientific evidence of conservative management of TF is limited and methodologically heterogeneous, with small sample sizes and varying outcome measures [21,23,24,25,26]. As highlighted by recent systematic reviews, the overall quality of evidence remains low, with no clear consensus on the most effective conservative strategy and a paucity of studies evaluating long-term outcomes or standardized protocols suitable for broader clinical application [23,24,25,26].

Often used in combination with other conservative approaches, transfer energy capacitive and resistive (TECAR) therapy has recently become popular as an alternative to surgical intervention for the treatment of various musculoskeletal disorders [27,28,29,30,31]. In particular, TECAR therapy has been reported to be effective in reducing pain intensity experienced by patients with chronic pain (i.e., knee, shoulder, hip, ankle, spine, and hand pain), as well as an effective method for improving bone, muscle, and joint injuries in athletes [30,32,33]. In detail, TECAR therapy represents a novel, non-invasive electrothermal modality that uses high-frequency electromagnetic waves (300 kHz–1.2 MHz) to generate energy transfer into target tissues and stimulate healing processes [34] by operating in two distinct treatment options—capacitive and resistive—which are designed to target superficial and deep tissues, respectively [29,33]. In capacitive mode, energy transmission generates heat in the superficial, low-impedance (water-rich) soft tissues such as adipose tissue, muscles, and cartilage, resulting in vasodilatation and tissue repair [35,36,37]. In the resistive mode, energy penetrates deeper tissue such as tendons, ligaments, and bones, and facilitates heat generation in dense (more resistant, low water content) structures, which enhances collagen synthesis, tissue regeneration, and reduces tissue inflammation [29,30,33].

Increasing evidence demonstrates the advantages of using TECAR therapy as a complementary treatment alongside common conservative physical therapies, helping to reduce recovery times from both acute injuries and chronic conditions [30,38,39,40,41,42,43]. Numerous studies have also explored the efficacy of TECAR therapy in reducing musculoskeletal pain (e.g., chronic low back pain, knee osteoarthritis, fatigue, and strain in runners) and alleviating painful inflammation caused by bone and joint disorders [30].

Despite its wide application in clinical practice and the encouraging evidence of preliminary findings showing its beneficial effects in pain relief and improved functional disability in different musculoskeletal conditions, the optimal therapeutic parameters and indications are yet to be fully established [33]. It is worth pointing out that current literature on TECAR therapy is marked by several limitations, including small sample sizes, variability in treatment protocols, and a lack of well-designed randomized clinical trials (RCTs), all of which hinder definitive conclusions regarding its clinical utility [30,33,40]. Moreover, most studies focus primarily on short-term outcomes and fail to investigate the long-term and sustained effects of TECAR therapy. While immediate improvements in pain, function, and muscle tone are frequently reported, the durability of these benefits remains uncertain in the absence of extended longitudinal follow-ups. This, in turn, is crucial in the context of chronic conditions, where ongoing management is pivotal to maintain patients’ quality of life [33]. Regarding the application of TECAR therapy for the management of tendinopathy, limited evidence is provided in the current literature [33,40,44].

Within this frame, to the best of the authors’ knowledge, the effects of combining TECAR therapy with splinting for TF patients have not been explored. Given their potentially complementary mechanisms of action—namely, TECAR’s role in promoting tissue microcirculation and pain relief, and splinting to prevent further tendon tissue inflammation or irritation, thereby promoting tissue healing—integrating these treatment modalities may enhance clinical outcomes. Therefore, this study aims to evaluate the relative effectiveness of combining splinting with resistive-mode TECAR therapy in the treatment of TF. We hypothesize that this combined approach will result in superior improvements in pain relief and functional recovery compared to either splinting or TECAR alone.

## 2. Materials and Methods

### 2.1. Study Design and Participants

This is a prospective, non-randomized clinical study that included all patients diagnosed with TF who referred to the Centre for Rehabilitative Medicine “Sport and Anatomy” of the University of Pisa, between March 2023 and October 2023. To ensure the consistency of the procedures, all interventions and assessments were performed in a single center.

Patients were included in the study if they were 18 years of age or older (both male and female), had a clinical diagnosis of TF, had experienced symptoms lasting for at least 4 weeks, and received no prior treatment for the same TF condition. The study included patients with one or more affected digits, including the thumb. Patients who experienced recent trauma to the affected hand, previous corticosteroid injections, and/or surgical intervention (open/percutaneous surgery) for the TF were excluded from the study. Also, patients diagnosed with diabetes mellitus were excluded from the study to minimize the potential confounding factors, as diabetic TF is typically characterized by greater clinical severity, higher recurrence, and reduced treatment responsiveness. Moreover, these patients often experience paresthesia or reduced cutaneous sensitivity, which could impair their perception of excessive heat during diathermy, thereby increasing the risk of skin injury/burns.

Subjects with Green grade IV were not included in the study, as conservative interventions have been reported to be ineffective for the locked digit and likely require surgical release [45,46,47].

### 2.2. Sample Size

An a priori power analysis was performed to determine the required sample size using G*Power software (version 3.1.9.6. *Düsseldorf, Germany*). Based on previous research, using an average of 2 as the minimal clinically important difference (MCID) for the pain scale, with significance set at α = 0.05, the power analysis indicated a sample size of n = 16 for 95% power.

### 2.3. Intervention

The study participants were allocated into one of the three groups, each corresponding to a different conservative treatment: (1) splinting group; (2) TECAR group; (3) TECAR combined with splint group. Group allocation was determined based on the availability of the TECAR device at the time of admission to therapy for each patient. No randomization and/or matching procedures were applied. Patients’ evaluations and treatments were performed by two experienced and certified Hand Therapists (C.M. and G.G.), specialized in hand and upper limb rehabilitation. The applied protocols are described below.

#### 2.3.1. Splinting Group

**Figure 1:**
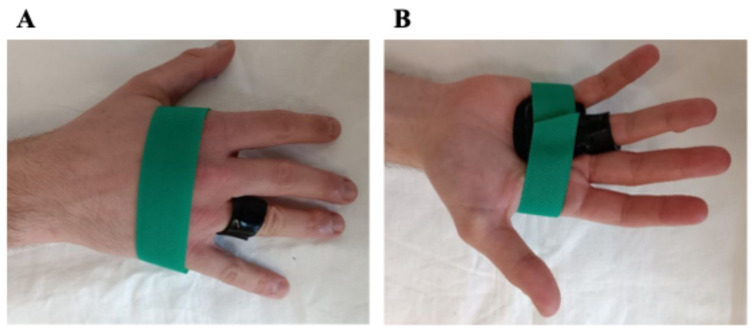
Dorsal (**A**) and volar (**B**) views of the splint.

At the initial visit, custom-made, thermoplastic splints (2.4 mm thick; Orfit Company, Intermedica, Milano, Italy) were manufactured for each patient, aiming to ensure maximum comfort and no pressure points. The design of the splint varied depending on whether the patient had a single TF, multiple TFs, or a TT. In detail, for a single TF, a custom-made ring splint with adjustable velcro was designed to hold the metacarpophalangeal (MCP) joint in a neutral position to allow partial active movement. In cases of multiple ipsilateral TFs, a volar orthosis with velcro was used to maintain the affected MCP joints in a neutral, resting position. Finally, for the TT, a circumferential “ring” splint was molded around the proximal phalanx of the affected finger to immobilize the proximal interphalangeal (PIP) joint. Once the participants and the therapists are satisfied with the fit of the splint, the subjects were instructed to wear the splint full-time, 24 h per day, for 8 weeks. The participants were also advised to avoid movements that could exacerbate their symptoms and to temporarily remove the splint only to perform some daily activities, primarily for hygiene purposes. In case of discomfort during use, pain or skin irritation, reduced finger swelling, and/or splint showing wear, during the follow-up evaluations, the splints were modified to provide the best and most comfortable fit (Figure 1).

#### 2.3.2. TECAR Group

**Figure 2:**
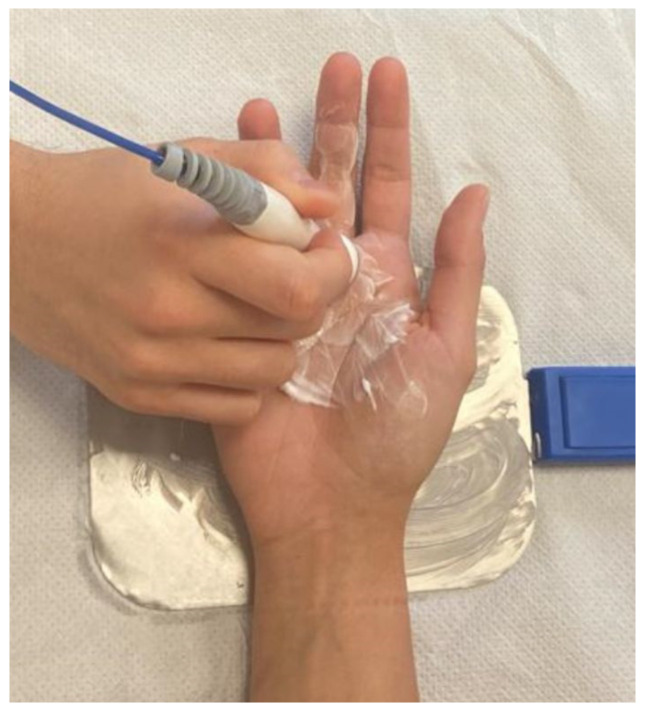
The position of the patient during TECAR therapy.

Since no standardized TECAR protocols for TF currently exist, a reproducible treatment protocol was developed. In detail, Tecar therapy was delivered using the VEGA device (BAC s.r.l., Florence, Italy), which incorporates both capacitive and resistive modes within a single bipolar electrode. Each treatment session lasted 20 min and was performed twice a week, for 8 weeks, which is consistent with previous literature [48,49,50,51]. The device was set in thermal mode, with power calibrated between 30 and 40% of the device’s maximum output, which allows to achieve deep heating while ensuring patient comfort and safety. The selected operating frequency (500 kHz) and intensity (power output) followed the manufacturer’s recommendations to generate mild, controlled diathermy without overheating the thin palmar soft tissues. The passive electrode was fixed on the dorsum of the hand, while the bipolar electrode was employed on the palmar side and moved by the operator along the flexor tendon path, with a special focus on the A1 pulley area. A layer of high-conductive cream was applied to facilitate the optimal distribution of endogenous heat therapy and effective contact between the electrode and the skin of the treated region. Both capacitive and resistive modes were employed sequentially within the same session. The capacitive mode was applied at the initial phase of treatment to warm the superficial soft tissues (rich in water and electrolytes, such as subcutaneous tissue, microvascular, and lymphatic networks), to reduce superficial inflammation, and facilitate local circulation in the treated area. Subsequently, the resistive mode was applied to target deeper and less hydrated structures with higher electrical resistance (such as tendons, ligaments, A1 pulley), to act specifically on the pathological interface between the flexor tendon and the A1 pulley, representing the primary site of mechanical friction in TF. In addition, as the device did not provide skin-temperature monitoring, safety was ensured by constant feedback gathered from the patients, operator palpation to detect excessive heating, and visual inspection of skin, to ensure a comfortable, warm sensation without causing skin discomfort or burning feeling or pain due to localized overheating (Figure 2).

#### 2.3.3. TECAR Combined with Splint Group

Participants in the combined TECAR + splinting group received the same splinting protocol described above, integrated with TECAR therapy.

### 2.4. Outcome Measurements

Experienced therapists conducted measurements at the baseline (before the intervention, T0), at 4th and 8th weeks after treatment initiation (T1 and T2, respectively), and at the one-month follow-up (T3). The primary outcome measures were pain intensity and Green’s classification to grade the severity of TF. Secondary outcomes included disability, overall improvement, and quality of life.

#### 2.4.1. Pain Intensity

Being a primary symptom of TF, pain reduction is often the primary goal of treatment. Pain was measured on an 11-point numerical rating scale (NRS), which quantifies the pain intensity experienced by the subject during the preceding day or the previous week [12]. Pain severity ranges from “0” (no pain) to “10” (worst conceivable pain).

#### 2.4.2. Green’s Classification to Grade the Severity of TF

The severity of the trigger digit was graded in accordance with Green’s classification [47,52] and reported in Table 1.

#### 2.4.3. QuickDASH

The Quick-Disabilities of the Arm, Shoulder, and Hand (QuickDASH) questionnaire was used to assess the impact of symptom severity and functional status on the subject’s ability to perform daily life activities [53,54]. The latter is a self-reported questionnaire consisting of 11 items that are related to patients’ symptom severity and ability to perform daily tasks. The cumulative score for all items is used to compute the scale score, which ranges from “0” (no disability) to “100” (highly severe disability).

#### 2.4.4. Trigger Finger Questionnaire for Clinical Outcomes Assessment

In accordance with previous studies [55,56,57], the evaluation of Severity of Triggering (ST), Frequency of Triggering (FT), and Functional Impact of Triggering (FIT) was based on a 0-to-10 point self-reported questionnaire for the assessment of TF, with higher scores indicative of worse symptoms of triggering (Table 2).

### 2.5. Statistical Analysis

The Kruskal–Wallis test, a non-parametric analysis of variance, was used to compare the differences between groups at the same time point for the following assessments: NRS, ST, FT, and FIT. The Friedman test was used to compare these assessments (NRS, ST, FT, and FIT) across time points, followed by post hoc pairwise comparisons performed using the Wilcoxon signed-rank test with Bonferroni correction.

A one-way ANOVA, a parametric analysis of variance, was used to analyze parametric demographic data at baseline and the results obtained from the QuickDASH questionnaire, followed by Student’s *t*-test for intra-group comparison. The Kolmogorov–Smirnov test was applied to assess the normal distribution of QuickDASH data.

Fisher’s exact test was used to compare non-parametric demographic data at baseline. For ordinal outcomes, such as the Green’s Score, data were summarized using the median and interquartile range (IQR) to account for their non-parametric nature.

For patients who had two fingers in the study, a Spearman correlation test was performed to determine if they could be treated as independent outcomes.

Statistical analyses were performed using StatView version 5.0.1.0 (SAS Institute Inc., Cary, NC, USA) and IBM SPSS Statistics (version 29.0.2.0. IBM Corp., Armonk, NY, USA). A *p*-value of < 0.05 was considered statistically significant. Data are presented as the mean ± SEM.

## 3. Results

### 3.1. Patient Characteristics

**Figure 3:**
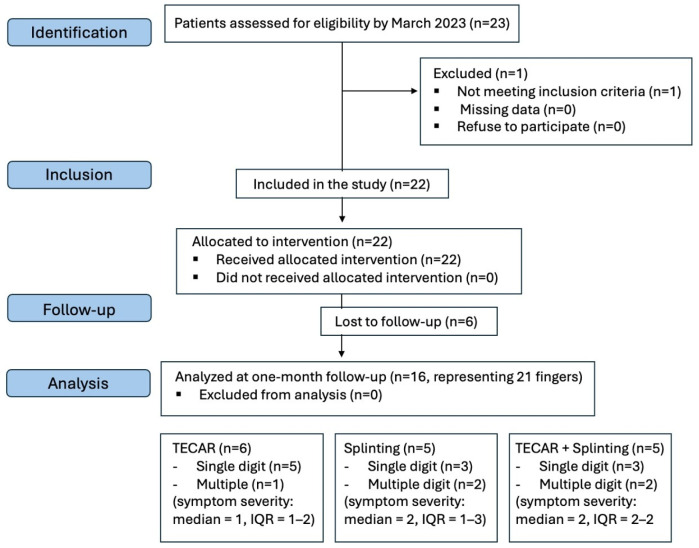
Flowchart of the study. Only subjects who attended their follow-up appointments were included in the final data analysis.

Of the eligible 23 patients (29 fingers) who were referred to our outpatient hand clinic, one patient (1 finger) was excluded from the study based on the predefined exclusion criteria; 6 patients (representing 7 trigger fingers) were excluded due to incomplete follow-up. The study involved 21 fingers from 16 subjects (8 females, 8 males; mean age 57.5 ± 15.8 years) who were able to comply with the entire treatment protocol and follow-up schedule (Figure 3), resulting in statistically homogeneous treatment groups.

The general characteristics of the study subjects at presentation are shown in Table 3. Of the 21 affected digits among these 16 subjects, 11 were on the dominant hand. Five patients had multiple TF; four patients had multiple (n = 2) TF in bilateral hands, while one patient had multiple (n = 2) TF on the very same dominant hand. The middle and ring fingers were more commonly involved than the other fingers.

No significant baseline differences were observed among groups regarding the age (*p* = 0.65), symptom duration (*p* = 0.66), severity (*p* > 0.05), affected side (dominant vs. non-dominant hand) (*p* > 0.05), and for the work status (employed vs. retired) (*p* > 0.05) (Supplementary Table S1). Of the five patients who had multiple trigger fingers, the Spearman correlation test did not find any correlation between the pain levels of the affected fingers (*p* = 0.36). Kolmogorov–Smirnov tests were performed on QuickDASH scores at baseline (T0) for the main treatment groups (TECAR + splinting vs. Splinting). No significant deviation from normality was observed (*p* = 0.5), supporting the use of parametric ANOVA for the analysis.

### 3.2. Primary Outcomes: Pain Intensity and TF Severity

**Figure 4:**
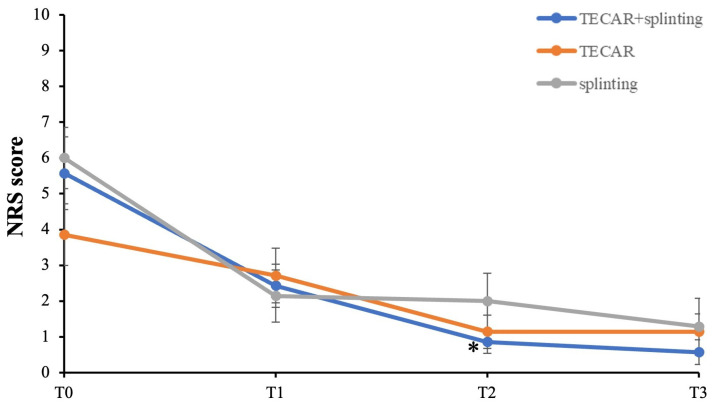
Primary outcomes with follow-up between groups. NRS score = pain intensity. * *p* < 0.05 vs. T0 TECAR + splinting.

The primary outcomes of the study were pain reduction and improvement in TF severity. Changes in clinical parameters across the three groups of intervention over time are reported in Figure 4. Compared to baseline, the results indicate that all the participants experienced improvement in pain at each of the three measured time points, although these changes did not reach statistical significance (Figure 4). Notably, it is worth pointing out that the combined treatment group (TECAR + splinting) demonstrated a significant and early reduction in pain relief at the second follow-up (T2) (*p* = 0.04, Cohen’s d = 1.74 large effect size), along with a trend toward greater improvement at the later time points compared to the single-treatment groups. This suggests that the combined treatment may offer more immediate and noticeable pain relief, while as time progresses, all three conservative approaches tend to converge, resulting in comparable outcomes for pain reduction.

### 3.3. Secondary Outcomes: Hand Functionality and Impact of Triggering

**Figure 5:**
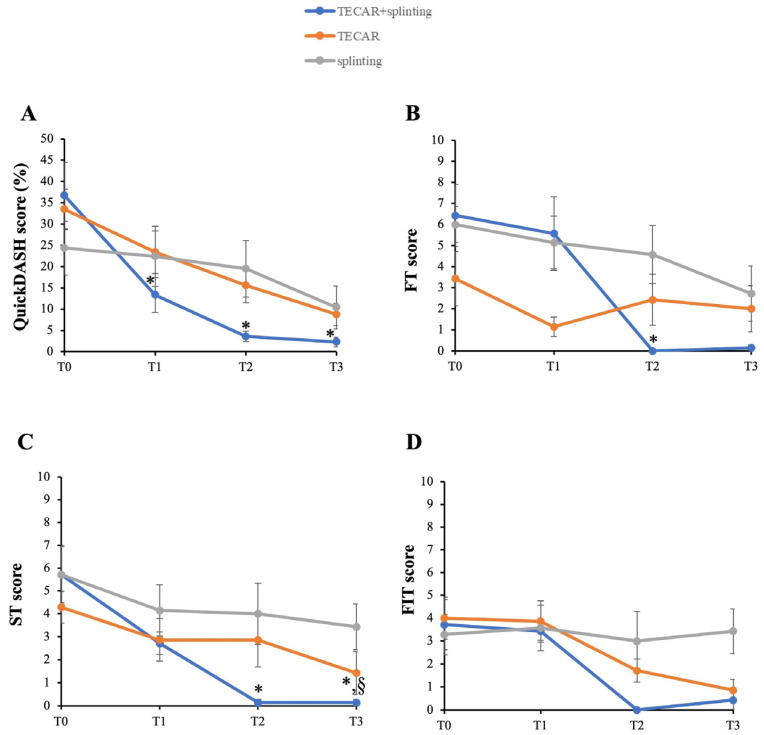
Secondary outcomes with follow-up between groups. (**A**) QuickDASH; (**B**) Frequency of triggering (FT); (**C**) Severity of triggering (ST); (**D**) Functional impact of triggering (FIT). * *p* < 0.05 vs. T0 TECAR + splinting; § *p* < 0.05 vs. other groups.

Results of the QuickDASH questionnaire regarding the assessment of the impact of symptom severity and hand functionality after the intended approach are reported in Figure 5A. While all groups showed improvements in QuickDASH scores compared to baseline, the combined intervention consistently resulted in greater and statistically significant changes at T1 and T2 (*p* = 0.03 and *p* = 0.002, respectively). Remarkably, this effect was maintained at the later follow-up time point (T3) (*p* = 0.002), nearly reaching a complete functional recovery in the combined treatment group.

In line with this, the assessment of frequency (FT) and severity (ST) demonstrated that the combined treatment significantly reduced these clinical parameters at T2 compared to T0 (Figure 5B and Figure 5C, respectively) (FT, *p* = 0.04; ST, *p* = 0.04). Moreover, the TECAR + splinting group showed a continued positive trend in ST improvement, with statistically significant differences at T3 (*p* = 0.04). Notably, at T3, the combined intervention became statistically significant compared to the single-treatment groups in terms of severity of triggering (*p* = 0.04). Noteworthy, physical examinations at each time point reveal no side effects with the splint use, TECAR therapy, or their combined application. In contrast, no significant differences were observed in FIT over time (Figure 5D).

## 4. Discussion

Trigger finger (TF), or stenosing tendovaginitis, is a common and debilitating hand condition that arises due to the combination of repetitive finger movements, local mechanical stresses, and inadequate healing processes [1,2,3,4,5]. These factors, in turn, can lead to tendon inflammation and a mismatch between the flexor tendon and its surrounding fibrous pulley, resulting in impaired smoothness of finger motion, pain, and difficulties in performing daily life activities [6,7,46,58].

Among first-line conservative treatments, the prescription of custom-made orthoses (i.e., splints) is generally regarded as an appropriate and effective tool to facilitate recovery in patients who prefer to avoid invasive procedures such as steroid injections [5,17,59]. However, there is a high variability among studies regarding the reported success rate with the use of splinting as primary intervention, ranging from 50% to 93% [46,47,55,60,61].

Noteworthy, given the multifactorial pathophysiology of TF, which involves both mechanical overload and inflammatory processes affecting the flexor tendon and the A1 pulley, some authors have investigated whether the addition of adjunct therapies to splinting could potentiate its clinical effects and accelerate recovery. For instance, a recent prospective study on the management of TF showed that combination therapy of splinting and topical NSAIDS or corticosteroid injection was more effective in preventing surgery than corticosteroid injection alone in patients with lower-grade TF [10,62]. Similarly, another randomized controlled trial demonstrated that wearing a static MCP joint splint for 3 months following a single injection of corticosteroid increased and stabilized the therapeutic benefits for TF [62]. Conversely, other studies found no significant differences in outcomes with the combined use of splinting plus steroid injection in all TF grades [63,64].

Within this frame, in recent years, the panel of rehabilitation options to optimize the management of TF patients has progressively expanded, incorporating new therapeutic approaches alongside the most traditional conservative ones, thus marking a shift towards multimodal, non-invasive rehabilitation strategies [7,65,66,67]. These adjunctive treatments include, for example, therapeutic ultrasound and extracorporeal shockwave therapy, which have emerged as promising non-invasive interventions for TF [7,21,68,69].

Within this rapidly evolving framework of therapeutic approaches for the management of musculoskeletal disorders, TECAR therapy has recently attracted considerable interest for its potential clinical benefits. Although widely adopted in clinical practice, high-quality clinical data supporting its efficacy remain limited [33,43]. While TECAR therapy shows promise in reducing pain and improving functional recovery across different musculoskeletal conditions, the existing literature is marked by small sample sizes, short-term follow-ups, and a lack of well-designed RCTs, which limit the statistical power and generalizability of findings [30,33,40]. Long-term efficacy and standardized treatment protocols also remain largely underexplored, especially for chronic conditions [33]. Nevertheless, recent literature reviews suggest that TECAR therapy may positively impact tendinopathies and soft-tissue healing by enhancing microcirculation, reducing inflammation, and promoting tissue regeneration [30,33,40,70].

Regarding tendon tissue, some previous studies have shown that TECAR therapy can modulate local blood flow and metabolic activity, contributing to accelerate recovery in various tendinopathies [28,30,32,33,34,44,48,71].

Given these promising beneficial effects, the present study investigated whether combining TECAR therapy with splinting could confer clinical advantages in TF patients in terms of pain relief and functional recovery. Compared to the splinting group, the combination of TECAR therapy plus splinting demonstrated earlier and more pronounced benefits in patients with varying degrees of TF according to Green’s classification. In particular, compared to single interventions, participants in the combined group experienced faster relief of pain and a quicker return to functional activities, as demonstrated by the greater reduction in NRS and QuickDASH scores at the earliest time point, namely after four weeks of therapy. These findings suggest that the combined treatment may offer slight advantages in the immediate follow-up periods for most parameters, related to improved overall quality of life. From a pathophysiological perspective, this is likely due to the TECAR-induced improvement in local tissue microcirculation and reduced tendon-pulley inflammation, which positively adds to the mechanical offloading provided by the splint. Moreover, improvements in TF severity, frequency, and functional impact of trigger were more sustained in the combined group at the end of the 8-week treatment and remained positive at one-month follow-up, although some differences did not reach statistical significance at T3. This, in turn, suggests that the combined therapy not only enhances immediate clinical outcomes but also provides longer-lasting effects on tendon functionality and patient-reported disability. Importantly, no adverse or side effects were reported with TECAR therapy, which is consistent with previous literature supporting its tolerability and safety as an adjunct non-invasive intervention for TF [30,33,40].

Although the present study provides preliminary evidence that combining TECAR therapy with splinting may offer additional early and sustained benefits in the short-term follow-up of TF patients, some study limitations and considerations should be noted. First, the small sample size and lack of blinding may limit the generalizability of the results and contribute to reduced homogeneity among the treatment groups. Second, potential comorbidities (e.g., osteoarthritis) and concomitant medication use (analgesics, NSAIDs) were not controlled for, which may have influenced outcome measures and treatment responses. Treatment compliance should also be considered a potential limitation, as adherence to the prescribed full-time splint regimen was based solely on patient self-report, which may not accurately reflect actual usage. Given these considerations, future studies on TECAR therapy for TF management should adopt a prospective, double-blind, randomized design, include stratification by Green’s grade and a broader representation of TF grades, incorporate longer follow-up periods, and use objective monitoring methods to assess splint adherence, to allow a more robust generalizability of findings. Finally, mechanistic studies aimed at elucidating the biological effects underlying the beneficial impact of TECAR therapy in TF are warranted.

## 5. Conclusions

To the best of the authors’ knowledge, this is the first report describing the effect of combined TECAR therapy plus splinting for the treatment of TF. Our findings suggest that integrating TECAR therapy with splinting may provide additional benefit for symptomatic management in TF patients, offering a balanced approach of pain relief, tendon healing, and functional recovery. However, this is an explanatory, hypothesis-generating study, and the findings should be interpreted with caution and not be considered definitive evidence of superiority. Randomized, blinded controlled trials are needed to confirm these preliminary observations and to provide further evidence of the effect.

## Figures and Tables

**Table 1:** Green’s classification to grade the severity of trigger finger.

Grade	Description
Grade I	Pain/history of catching
Grade II	Demonstrable catching, but can actively extend the digit
Grade III	Demonstrable locking, requiring passive extension
Grade IV	Fixed flexion contracture

**Table 2:** Questionnaire for the assessment of triggering.

Severity of Triggering (ST)	Score
Lock, must manually pull out finger	10
Lock, can extend finger actively	8
Click with intense pain	5
Click with discomfort, no pain	3
Click with no discomfort or pain	1
No click, no discomfort	0
**Frequency of Triggering (FT)**	**Score**
All of the time	10
Several times per day	8
Once daily	6
Several times per week	4
About once per week	2
Not every week	1
Never	0
**Functional Impact of Triggering (FIT)**	**Score**
Disability (unable to perform any task with hand)	10
Severe (unable to perform 75% of tasks)	8
Moderate (unable to perform 50% of tasks)	6
Minimal (able to perform, but with discomfort)	3
None	0

**Table 3:** Demographic background of the entire sample (n = 21 digits/16 subjects).

Variable	Value
Age (years) ± SD	57.5 ± 15.8
Female (%)	8 (50%)
Dominant hand affected (%)	11 (68.7%)
Affected digit (%)	-
Thumb	4 (19.0%)
Index finger	2 (9.5%)
Middle finger	5 (23.8%)
Ring finger	8 (38.1%)
Little finger	2 (9.5%)
Work status (%)	-
Employed	9 (56.2%)
Unemployed	0 (0%)
Retired	7 (43.7%)

## Data Availability

The data supporting the conclusions of the current study are available from the corresponding author upon reasonable request.

## Supplementary Materials

The following supporting information can be downloaded at: https://www.mdpi.com/article/10.3390/life16010030/s1, Table S1.

## Abbreviations

The following abbreviations are used in this manuscript:

FT	Frequency of Triggering
FIT	Functional Impact of Triggering
MCP joint	Metacarpophalangeal joint
NRS	Numerical rating scale
NSAIDs	Nonsteroidal anti-inflammatory drugs
PIP joint	Proximal interphalangeal joint
QuickDASH	Quick-Disabilities of the Arm, Shoulder, and Hand
ST	Severity of Triggering
TECAR	Capacitive and Resistive Electric Transfer Therapy
TF	Trigger finger
TT	Trigger thumb
