# Supplementary material for: Short-Term Effects of Capacitive and Resistive Electric Transfer Therapy and Static Splinting in the Management of Trigger Finger: A Non-Randomized Clinical Study
Source: Life (Basel). 2025 Dec 25;16(1):30. doi: 10.3390/life16010030 (PMC12843178; doi:10.3390/life16010030)
Supplement: Supplementary file 1 [file life-16-00030-s001.zip › life-4014237-supplementary.pdf]

Supplementary Table 1

	Severity (p-value)	Dominant vs non-dominant (p-value)	Employed vs retired (p-value)
**Tecar+splinting vs Tecar**	0.5	0.13	0.43
**Tecar+splinting vs Splinting**	0.1	0.37	0.42
**Tecar vs Splinting**	0.25	0.25	0.3

At baseline, pairwise comparisons between the groups using Fisher's exact test did not reveal significant differences in the distribution of finger trigger severity. Therefore, the groups can be considered comparable at the start of the study, and the post-treatment analysis evaluates the effect of the interventions starting from a similar baseline condition across groups.
